# Accelerating Protein Docking in ZDOCK Using an Advanced 3D Convolution Library

**DOI:** 10.1371/journal.pone.0024657

**Published:** 2011-09-19

**Authors:** Brian G. Pierce, Yuichiro Hourai, Zhiping Weng

**Affiliations:** 1 Program in Bioinformatics and Integrative Biology, University of Massachusetts Medical School, Worcester, Massachusetts, United States of America; 2 Computational Biology Research Center (CBRC), National Institute of Advanced Industrial Science and Technology (AIST), Tokyo, Japan; Koç University, Turkey

## Abstract

Computational prediction of the 3D structures of molecular interactions is a challenging area, often requiring significant computational resources to produce structural predictions with atomic-level accuracy. This can be particularly burdensome when modeling large sets of interactions, macromolecular assemblies, or interactions between flexible proteins. We previously developed a protein docking program, ZDOCK, which uses a fast Fourier transform to perform a 3D search of the spatial degrees of freedom between two molecules. By utilizing a pairwise statistical potential in the ZDOCK scoring function, there were notable gains in docking accuracy over previous versions, but this improvement in accuracy came at a substantial computational cost. In this study, we incorporated a recently developed 3D convolution library into ZDOCK, and additionally modified ZDOCK to dynamically orient the input proteins for more efficient convolution. These modifications resulted in an average of over 8.5-fold improvement in running time when tested on 176 cases in a newly released protein docking benchmark, as well as substantially less memory usage, with no loss in docking accuracy. We also applied these improvements to a previous version of ZDOCK that uses a simpler non-pairwise atomic potential, yielding an average speed improvement of over 5-fold on the docking benchmark, while maintaining predictive success. This permits the utilization of ZDOCK for more intensive tasks such as docking flexible molecules and modeling of interactomes, and can be run more readily by those with limited computational resources.

## Introduction

Interactions between biomolecules are crucial to the function of biological systems, forming the basis of normal and aberrant cellular behavior, as well as defense against external pathogens. To fully understand these interactions, atomic-level descriptions of the structures of their binding interfaces are essential. While the structures of many protein-protein complexes have been characterized experimentally via x-ray crystallography and deposited in the Protein Data Bank (PDB; [1]), the majority of known complexes have not, providing an opportunity for predictive computational techniques to help elucidate these structures. Molecular docking approaches, which take two (or more) structures as input and predict the structure of their complex, are increasingly being used for this purpose [2].

The success of protein-protein docking algorithms in the past decade has given rise to several exciting developments in the field. This includes addressing molecular flexibility during binding by “cross-docking” ensembles representing snapshots of mobile structures [3], [4], [5], or combining docking results of rigid (or semi-rigid) substructures [6], [7]. On a larger scale, other recent work includes the application of protein-protein docking to predict the structure of the yeast interactome [8], and the use of protein docking to distinguish binding versus nonbinding proteins based on docking scores [9]. These areas of progress indicate that faster and more efficient docking algorithms are key to helping improve both predictive accuracy and proteomic coverage.

Previously our laboratory developed the program ZDOCK, which uses a grid-based representation of two proteins and a 3-dimensional (3D) fast Fourier transform (FFT) to efficiently explore the rigid-body search space of docking positions [10]. The most recent version, ZDOCK 3.0, has a scoring function that includes shape complementarity, electrostatics, and a pairwise atomic statistical potential developed using contact propensities of transient protein complexes [11]. ZDOCK 3.0 showed vast improvements in its predictive ability versus the previous version when tested on a protein-protein docking benchmark [11], and has led to highly successful performance in the blind protein docking experiment, CAPRI [12], [13]. However, with the improved accuracy due to the pairwise statistical potential, the running time and memory usage of ZDOCK increased significantly, as seven FFTs (rather than two in the previous version, ZDOCK 2.3 [14]) needed to be computed per docking orientation.

To reduce this computational burden and make proteomic scale docking and ensemble docking approaches more tractable, we have developed a new version of ZDOCK that retains the predictive accuracy of ZDOCK 3.0 while vastly improving its computational performance. This was achieved by integrating an FFT library that was designed to improve 3D FFT performance [15], as well as several improvements to the molecular discretization to further reduce the grid size required to represent the input proteins. These optimizations were evaluated against 176 test cases in a newly released version of a protein docking benchmark [16], resulting in over 8.5-fold average improvement in running time. We also implemented these updates on ZDOCK 2.3, for those users who have pipelines or protocols in place with this tool (e.g. protein/DNA docking [17]), which resulted in 5.5-fold improvement in running time. Examining the test cases with the highest levels of running time improvement showed that the primary factor in improving performance is the reduced grid size due to the new FFT library.

## Methods

### ZDOCK Overview

The ZDOCK algorithm, which followed from initial efforts in FFT-based protein docking [18], [19] and was described in detail by Chen and Weng [10], includes the following steps (not including the pre-processing step of marking surface atoms and atom types in PDB files). The terms “receptor” and “ligand” refer to the two input proteins, with the receptor generally being the larger protein or known to function as a receptor *in vivo* (e.g. an antibody in an antibody/antigen interaction).

**ZD1. Center receptor coordinates at origin based on center of mass.**

**ZD2. Center ligand coordinates at origin based on center of mass.**

**ZD3. Select cubic grid size to contain centered molecules for FFT.**

ZD4. Discretize receptor, assigning scores to 3D grid(s) of complex numbers.

ZD5. Rotate input ligand to random orientation, if specified.

ZD6. Rotate ligand to Euler angles from uniformly distributed set, and discretize.

**ZD7. Perform 3D FFT to compute convolution between ligand and receptor grids, and select top scoring position from the resultant grid.**

ZD8. Repeat steps 6–7 for a total of 3,600 ligand rotations (15° angular sampling) or 54,000 ligand rotations (6° angular sampling).

Here we present major improvements to ZDOCK's initial orientation and FFT procedures (bold steps above), while not modifying the discretization protocols that embody the ZDOCK scoring function. Previous ZDOCK versions and their scoring terms include: ZDOCK 1.3 [10]: Grid-based shape complementarity, atomic contact energy (ACE; [20]), electrostatics: ZDOCK 2.1 [21]: Pairwise shape complementarity (PSC); ZDOCK 2.3 [14]: PSC, ACE, electrostatics; ZDOCK 3.0 [11]: PSC, interface atomic contact energy (IFACE), electrostatics.

### ZDOCK Modifications

Modification of ZDOCK to improve its efficiency consisted of the following successive improvements:

1. **Integrating the Conv3D library.** This entailed replacing the standard FFT library calls (FFTW for Linux/MacOS, ESSL for IBM platforms) for performing 3D convolution. Previously, ZDOCK discretized the receptor and ligand (the two input proteins) onto two cubic (NxNxN, where N is the dimension of each side) 3D grids. The Conv3D library, which is based on FFTW (http://www.fftw.org), and is optimized specifically for high efficiency convolution of 3D molecular structures [15], effectively allows for non-cubic rectangular grids by performing a series of 1D FFT operations that skip empty rows of the 3D cube. With Conv3D, we selected a rectangular 3D grid to contain the receptor, which is fixed during the ZDOCK run, while for the ligand (which is rotated through a series of Euler angles during ZDOCK) a cubic grid was maintained, to allow for a constant grid size through successive ligand rotations. In addition, the Conv3D library uses single rather than double precision (the latter of which is default in FFTW and used by ZDOCK previously), which required minor modifications to the ZDOCK code. This change in decimal representation, which helped improve memory and computational time, led to minor changes in scores for some test cases between ZDOCK runs, but no significant change in success rate.
2. **Optimal centering of the input proteins.** We further optimized ZDOCK by reducing the grid sizes of the receptor and ligand through careful selection of the molecular centers for grid placement. Prior to discretization onto the grid in ZDOCK, the input receptor and ligand are translated from their original coordinates to the origin (Steps ZD1 and ZD2 above). This translation was formerly selected using the molecules' centers of mass, but this can be non-optimal, particularly where a protein's mass distribution is not even. In the current ZDOCK implementation, we compute the receptor's maximal dimensions along each Cartesian axis and use the halfway points in each dimension for its grid center. For the ligand, which rotates through a series of angles during ZDOCK, we calculated its center as the origin of a bounding sphere using the algorithm proposed by Ritter [22]. This attempts to calculate the origin and radius denoting a sphere that encompasses all points of the ligand within a sphere of minimal size. As a minimal bounding sphere is not guaranteed through this algorithm, the bounding sphere radius from Ritter's algorithm was compared to the radius for the center of mass (i.e. the greatest distance of any atom from the center of mass), and the method (bounding sphere or center of mass) giving the minimum radius was selected for centering the ligand.
3. **Rotation of the receptor.** Another improvement of ZDOCK is the use of a rotated receptor with respect to its input angular orientation. This gives improved efficiency versus a receptor with an input rotation that would leave excess unoccupied grid points when discretized onto a rectangular grid. To select this receptor rotation, ZDOCK iterates through 3,600 evenly distributed Euler angles (the same as those used by ZDOCK to rotate the ligand in step ZD6 above, provided by Dr. Julie C. Mitchell) and evaluates the grid size required for each rotated receptor. The angle with the minimum required grid size is then chosen for input receptor rotation.
4. **Switching of the ligand and receptor.** In the past, ZDOCK has kept the user-specified receptor fixed during ZDOCK execution, while rotating the ligand and re-discretizing it for each rotational angle. In the current ZDOCK version, this has been modified so that the receptor and ligand can be switched dynamically during ZDOCK execution. This switch is evaluated at the beginning of ZDOCK execution, by calculating the minimum grid required by the switched receptor and ligand (rotating the ligand through the 3,600 angles as described above), and comparing with the grid size from the original receptor and ligand. Based on the grid size comparison, either the original receptor will be fixed, or the ligand and receptor will be switched so that the ligand will be fixed and bounded by a rectangular grid.

### ZDOCK Implementation

We previously released ZDOCK 2.3 and 3.0 with the Conv3D library (Step 1) as interim versions, named ZDOCK 2.3.1 and ZDOCK 3.0.1 respectively. The current releases include Steps 1–4 and are ZDOCK 2.3.2 and ZDOCK 3.0.2. Due to the input rotation of the receptor and the possible switching of the ligand and receptor, the ZDOCK output file format required slight modification of its header format, and consequent updating of the code to create ZDOCK predictions (create_lig) from the output file. However, the create_lig program still generates structural predictions with the receptor fixed with respect to the coordinates of the input PDB file, so that the internal optimization of the receptor and ligand coordinates for ZDOCK discretization are not visible to the end user.

We also introduced a command line flag in ZDOCK (“-F”) to provide users the option of keeping the input receptor fixed (not rotated or switched with the ligand) during ZDOCK execution, resulting in the same ZDOCK output file format as previous versions. This entails Steps 1 and 2 (without Steps 3 and 4), and can be used for improved performance versus the base ZDOCK version (2.3 or 3.0) when users require the original ZDOCK output file format for their post-processing pipeline.

### Figures

Data plots were generated using gnuplot (http://www.gnuplot.info), and molecular structures were visualized using PyMOL (http://www.pymol.org).

## Results

### Computational Performance

After updating ZDOCK versions 3.0 and 2.3 with the Conv3D FFT library and improved molecular representation (detailed in the Implementation section), we tested these new versions (3.0.2 and 2.3.2) for their computational efficiency using all 176 unbound test cases of protein-protein docking Benchmark 4.0 [16]; results are given in Table 1. Each run of ZDOCK used default angular sampling (3,600 ligand rotations), and a single 2.8 GHz 64-bit Opteron processor with 8 GB available RAM. To test the improvements due to specific modifications, we also measured the performance of ZDOCK with Conv3D only (Step 1 in Implementation; 3.0.1 and 2.3.1), and Conv3D with improved centering (Steps 1 and 2; 3.0.2f and 2.3.2f).

**Table 1:** Average running time, running time fold improvement, and memory usage of optimized ZDOCK versions.

Name	Optimization^1^	Running Time (min)	Fold Improvement^2^	Memory (MB)
ZDOCK 3.0	-	167.1	-	700
ZDOCK 3.0.1	Conv3D	26.5	6.4	303
ZDOCK 3.0.2f	Conv3D+Cent	23.2	7.2	282
**ZDOCK 3.0.2**	**Conv3D+Cent+Rot+Switch**	**18.9**	**8.6**	**256**
ZDOCK 2.3	-	53.2	-	296
ZDOCK 2.3.1	Conv3D	13.1	4.0	215
ZDOCK 2.3.2f	Conv3D+Cent	11.2	4.7	203
**ZDOCK 2.3.2**	**Conv3D+Cent+Rot+Switch**	**9.3**	**5.5**	**191**

All values are averages from running ZDOCK on 176 unbound docking test cases, each run using a single 2.8 GHz 64-bit Opteron processor with 8 GB available RAM. Bold rows correspond to the fully optimized ZDOCK versions.

1 Optimization scheme. Conv3D = new 3D FFT library, Cent = optimal receptor centering, Rot = optimal receptor rotation, Switch = switch ligand and receptor. See Implementation section for details.

2 Average fold improvement in running time versus the previous major ZDOCK version (3.0 or 2.3).

The most dramatic improvements were seen for ZDOCK 3.0.2, with 18.9 minutes average running time for the docking benchmark, from an original average running time of 167.1 minutes. This is nearly three times less than the average running time for ZDOCK 2.3 on the docking benchmark. On average, this version had an 8.6-fold improvement in running time versus ZDOCK 3.0; this was significantly higher than the 6.4-fold improvement from Conv3D alone (ZDOCK 3.0.1), though integrating Conv3D was evidently responsible for the majority of the running time improvement. Required memory concomitantly was reduced for these ZDOCK improvements, with less than half of the memory for ZDOCK 3.0 required, on average, by ZDOCK 3.0.2 (256 MB, versus 700 MB for ZDOCK 3.0).

Mirroring the improvements for ZDOCK 3.0, the running time and memory usage of ZDOCK 2.3 were also improved via these library and discretization modifications, although to a lesser extent (5.5-fold versus 8.6-fold improvement in running time). While ZDOCK 2.3 has over three times faster average running time on the docking benchmark versus ZDOCK 3.0, ZDOCK 2.3.2 is still over twice as fast as ZDOCK 3.0.2.

### Docking Success and Hit Count

**Figure 1:**
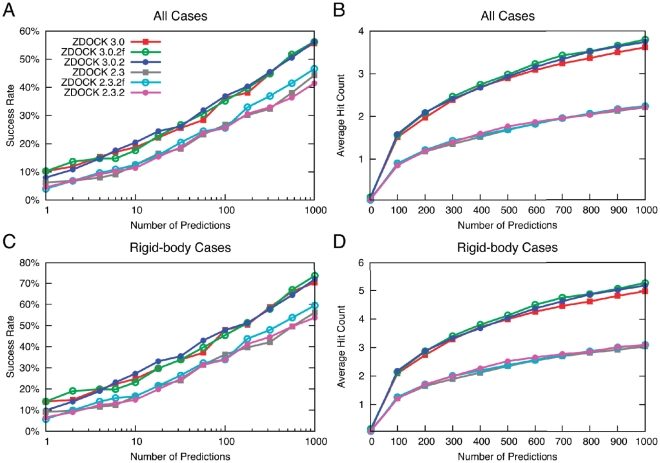
Success rate and hit count of original and optimized versions of ZDOCK for all test cases (A, B) and rigid-body test cases (C, D) of docking Benchmark 4.0. ZDOCK 3.0.2f and 2.3.2f represent the optimized versions with receptor rotation and receptor/ligand switching turned off, while ZDOCK 3.0.2 and 2.3.2 represent the fully optimized versions of ZDOCK 3.0 and ZDOCK 2.3, respectively. Success rate is defined as the percentage of cases with hits (RMSD less than or equal to 2.5 Å when comparing with Cα atoms in the bound interface to corresponding Cα atoms in the prediction) for a given number of top-ranked ZDOCK predictions per test case, while hit count is the average number of hits across the set of cases for a given number of top-ranked ZDOCK predictions per test case.

To ensure that the predictive accuracy of ZDOCK was maintained during optimization, we measured the success rates and the average number of hits for the original and updated versions of ZDOCK (Figure 1). As before, hits are defined as predictions with interface Cα root mean square distance (RMSD) ≤2.5 Å from the bound structure, and framework regions of antibodies were blocked prior to docking, to avoid non-CDR binding predictions as described previously [10]. Details of the ZDOCK results are given in Tables S1 and S2.

As shown on a previous version of the docking benchmark [11], the success rate for ZDOCK 3.0 and hit count are substantially higher than for ZDOCK 2.3; this is also seen for the new ZDOCK implementations presented here (ZDOCK 3.0.2f and ZDOCK 3.0.2 versus ZDOCK 2.3.2f and ZDOCK 2.3.2). These new versions of ZDOCK have approximately the same success rates of the respective previous versions, with some minor differences (e.g. higher success at N = 2 for ZDOCK 3.0.2f) that appear insignificant and not sustained for varying numbers of predictions. Hit counts (Figure 1B and 1D) likewise follow similar trends as the previous ZDOCK versions; although there are slightly higher hit counts for ZDOCK 3.0.2f and ZDOCK 3.0.2 versus ZDOCK 3.0 at larger numbers of predictions, this is much smaller and less significant than the differences between ZDOCK 2.3 and ZDOCK 3.0. For the rigid-body cases (121 out of 176 cases; Figure 1C and 1D), there is an upward shift in success rate and hit count compared with the results for all test cases (Figure 1A and 1B), which is to be expected given the lower binding conformational changes of these cases on average. However, considering just the rigid-body cases does not yield any differences in the relative docking success between the ZDOCK versions.

### Computational Performance Details

**Figure 2:**
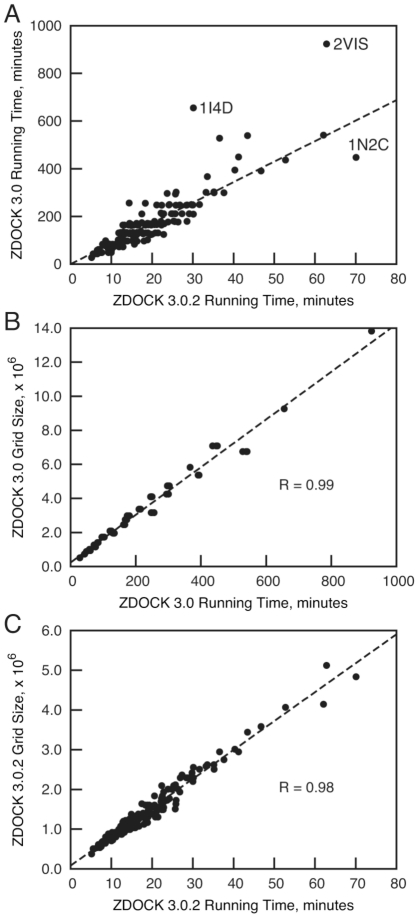
ZDOCK running time and grid size comparison for all 176 Benchmark 4.0 test cases. (A) ZDOCK 3.0 running time versus ZDOCK 3.0.2 running time on a single 2.8 GHz processor; outlier test cases 2VIS, 1I4D, and 1N2C are labeled. (B) ZDOCK 3.0 grid size versus ZDOCK 3.0 running time for all test cases. Grid size is the product of the number of points in each dimension of the cubic grid, calculated based on the sum of the cubic grids calculated for the ligand and receptor individually. (C) ZDOCK 3.0.2 grid size versus ZDOCK 3.0.2 running time for all test cases. Grid size is the product of the number of grid points in each dimension, N_x*N_y*N_z, where N_x is calculated using the number of grid points for the receptor in the x dimension plus the number of points of the ligand grid in the x dimension (which is equal to the number of points for the y and z dimensions as the ligand is in a cubic grid), and likewise for the other dimensions N_y and N_z.

To examine the extent of the running time improvement among individual cases, we compared running times for ZDOCK 3.0 with ZDOCK 3.0.2 for all 176 Benchmark 4.0 test cases (Figure 2A; running time details are given in Tables S3 and S4 for ZDOCK 2.3.2 and 3.0.2 respectively). Most cases follow the trend of 8.6-fold average improvement, with the exception of a few outlier points. This includes 2VIS and 1I4D, which showed greater-than-average improvement of 14.7-fold and 21.7-fold in running time, respectively, and 1N2C, which was below the average but still had a substantial 6.4-fold improvement in running time. The minority of cases (46 out of 176) had ligand and receptor switched by ZDOCK 3.0.2 (Table S3), and some of these (e.g. 2VIS) clearly had dramatic improvements in running time. The high correlation between grid size and running time is shown in Figures 2B (ZDOCK 3.0; R = 0.99) and 2C (ZDOCK 3.0.2; R = 0.98). This indicates that the efficient use of the grid search space by ZDOCK 3.0.2 provides the basis for the improvements in running time across the docking benchmark.

Also evident is the clustered nature of running times for ZDOCK 3.0, as seen in the horizontal bands in Figure 2A and the overlapping points in Figure 2B (making the plot appear sparser than Figure 2C). This is due to the cubic grid size in ZDOCK 3.0 being selected from a finite set of numbers (as specified by FFTW, or ESSL for IBM ZDOCK compilations), which in turn leads to similar running times between cases that share the same grid size. With Conv3D, the receptor grid size is selected from a finite set of numbers for each x, y, and z dimension, leading to more possible 3D grid sizes available and a consequent dispersion of running times versus those for ZDOCK 3.0.

**Figure 3:**
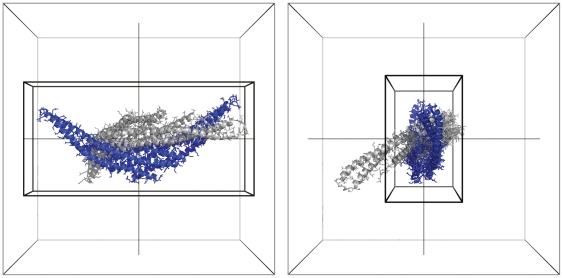
Grid sizes and initial rotation of the receptor of test case 1I4D, front (lengthwise) view (left) and side view (right). The outer (gray) box represents the grid for the receptor given by ZDOCK 3.0 and the gray molecule represents its corresponding centered orientation, while the blue molecule and black box represent the rotated and centered molecule and grid for ZDOCK 3.0.2. Grid dimensions for ZDOCK 3.0 are 140×140×140 = 2,744,000 cells, while the dimensions for ZDOCK 3.0.2 are 40×66×134 = 353,760 cells.

An individual example of a test case with dramatic speed improvement is shown in Figure 3, which shows the structure of the 1I4D receptor and its representative grids for ZDOCK 3.0 and 3.0.2. Is clear that the ability to use a rectangular rather than a cubic grid to represent this elongated protein, along with optimal alignment along the x, y, and z axes, enables the vast improvement in efficiency for this test case. On the other hand, 1N2C, which has below-average improvement as noted above, has two globular symmetric proteins as receptor and ligand, offering less opportunity for optimizing docking speed via grid size reduction or rotation of an input molecule.

## Discussion

These new versions of ZDOCK can be utilized more readily by those with limited computing resources, as well as those who are addressing challenging areas at the forefront of structural prediction, such as molecular flexibility and 3D network modeling. In fact, ZDOCK 3.0 (which has the same scoring function as ZDOCK 3.0.2, without performance optimization) was used to predict the structure of the yeast interactome using a large supercomputing cluster [8]. We hope that the improved efficiency in ZDOCK 3.0.2 will permit further utilization of this docking tool in advanced research efforts. Structural interactome modeling in particular has had numerous recent advances [23], and rigid-body docking of domains and proteins from structural genomics efforts can complement atomic-level interactions modeled based on homology, and build upon the success in modeling structures of sub-proteome interaction networks [24].

Others have recently presented algorithms that perform fast rigid-body protein docking using FFT approaches. Notably, the program Hex, which uses spherical harmonics rather than a 3D Cartesian grid to represent proteins, but does not contain atomic pairwise potential terms as in ZDOCK 3.0.2, was optimized using graphical processors to achieve very high docking efficiency [25]. In the same study, comparison of ZDOCK 3.0.1 with the program PIPER (which has pairwise potential terms based on docking decoys [26]) found ZDOCK 3.0.1 to be over 50 times faster when both are run on a single CPU of the same speed.

Future work will include the development and validation of ensemble and cross-docking approaches using ZDOCK 3.0.2, as well as incorporating it into the ZDOCK server (http://zdock.bu.edu), which at present uses ZDOCK 2.3 as the computational requirements of ZDOCK 3.0 precluded its use in the server framework.

By incorporating several methods to improve computational efficiency into the ZDOCK program, we have achieved significant performance gains for two versions of the ZDOCK program, with no loss in docking accuracy. The two new versions of ZDOCK presented here, 2.3.2 and 3.0.2, are freely available to academic and non-profit users at: http://zlab.umassmed.edu/zdockconv3d/.

## Supporting Information

Table S1 Predictive performance of ZDOCK 2.3, 2.3.1, 2.3.2f and 2.3.2 for the test cases in Benchmark 4.0.
(PDF)

Table S2 Predictive performance of ZDOCK 3.0, 3.0.1, 3.0.2f and 3.0.2 for the test cases in Benchmark 4.0.
(PDF)

Table S3 Running times of ZDOCK versions 2.3, 2.3.1, 2.3.2f, and 2.3.2 for the test cases in Benchmark 4.0.
(PDF)

Table S4 Running times of ZDOCK versions 3.0, 3.0.1, 3.0.2f, and 3.0.2 for the test cases in Benchmark 4.0.
(PDF)
