# Supplementary material for: Accelerating Protein Docking in ZDOCK Using an Advanced 3D Convolution Library
Source: PLoS One. 2011 Sep 19;6(9):e24657. doi: 10.1371/journal.pone.0024657 (PMC3176283; doi:10.1371/journal.pone.0024657)
Supplement: Table S1 — Predictive performance of ZDOCK 2.3, 2.3.1, 2.3.2f and 2.3.2 for the test cases in Benchmark 4.0. (PDF) [file pone.0024657.s001.pdf]

Table S1. Predictive performance of ZDOCK 2.3, 2.3.1, 2.3.2f and 2.3.2 for the test cases in Benchmark 4.0. Hits2K denotes the number of hits in the top 2000 predictions, Rank is the rank of first hit, and RMSD is the RMSD of first hit

	ZDOCK 2.3			ZDOCK 2.3.1			ZDOCK 2.3.2f			ZDOCK 2.3.2		
**Test Case**	**Hits 2K**	**Rank**	**RMSD**	**Hits 2K**	**Rank**	**RMSD**	**Hits 2K**	**Rank**	**RMSD**	**Hits 2K**	**Rank**	**RMSD**
1AHW	6	12	1.62	7	13	1.62	8	20	1.96	6	39	1.88
1BVK	1	802	2.42	1	787	2.42	0	2175	1.93	0	2699	2.02
1DQJ	1	1141	2.34	1	1141	2.34	0	--	--	1	1922	2.45
1E6J	12	19	1.60	12	19	1.60	9	17	1.70	15	10	1.34
1JPS	3	93	1.78	3	85	1.78	4	46	0.85	4	12	1.40
1MLC	6	9	1.03	6	10	1.03	11	46	1.86	11	11	1.60
1VFB	2	1189	1.89	2	1187	1.89	1	545	2.17	2	205	1.83
1WEJ	7	34	0.79	7	33	0.79	8	103	1.20	11	338	1.93
2FD6	3	698	2.03	3	678	2.03	2	224	2.16	0	2120	1.76
2I25	5	345	1.59	5	337	1.59	3	60	2.09	5	46	1.82
2VIS	5	575	2.39	5	552	2.39	6	272	2.14	7	500	1.96
1BJ1	23	8	1.04	23	8	1.04	18	2	0.87	23	4	0.87
1FSK	16	1	0.94	16	1	0.94	14	1	0.98	14	1	0.89
1I9R	6	13	2.22	6	13	2.22	4	34	2.03	5	5	2.38
1IQD	8	54	1.02	9	53	1.02	3	15	2.21	9	125	2.15
1K4C	4	1159	2.26	4	1176	2.26	4	680	1.66	4	93	1.14
1KXQ	4	93	1.22	4	93	1.22	6	115	1.46	4	105	1.28
1NCA	8	12	0.94	8	12	0.94	9	13	1.24	8	3	1.85
1NSN	2	589	1.78	2	592	1.78	2	422	2.48	0	2519	1.94
1QFW	4	56	1.49	4	58	1.49	7	22	1.53	2	499	1.56
2QFW	5	41	1.94	5	41	1.94	6	132	2.19	7	130	2.09
2JEL	8	391	2.39	8	407	2.39	10	25	2.05	10	33	1.34
1AVX	3	217	1.68	3	212	1.68	6	143	1.77	8	109	2.43
1AY7	0	2265	2.20	0	2410	2.20	1	1219	1.71	2	733	2.16
1BVN	10	35	1.13	10	35	1.13	8	30	1.70	9	31	1.25
1CGI	2	712	2.48	2	703	2.48	1	1253	2.36	0	--	--
1CLV	13	9	2.41	13	9	2.41	10	33	2.12	10	11	2.43
1D6R	1	1104	2.13	1	1112	2.13	1	1833	2.04	2	1143	2.02
1DFJ	7	1	2.16	7	1	2.16	6	3	2.25	7	1	2.03
1E6E	8	161	2.00	8	154	2.00	7	6	1.96	5	135	2.27
1EAW	15	3	1.06	16	3	1.06	19	3	1.60	20	2	1.37
1EWY	7	91	2.07	7	94	2.07	4	155	1.96	5	57	2.24
1EZU	0	--	--	0	--	--	3	1160	1.58	3	1085	2.29
1F34	2	18	1.57	2	17	1.57	1	6	2.00	1	19	1.80
1FLE	6	95	2.12	6	95	2.12	4	52	2.16	7	15	2.15
1GL1	1	1466	2.14	1	1459	2.14	2	1289	2.48	4	218	2.38
1GXD	1	1490	1.79	1	1486	1.79	0	--	--	0	--	--
1HIA	0	--	--	0	--	--	0	--	--	0	--	--
1JTG	12	1	1.59	12	1	1.59	11	1	1.33	9	1	1.60
1MAH	10	2	1.52	12	2	1.52	15	1	1.52	11	2	1.43
1N8O	6	1	1.26	6	2	1.26	8	11	2.18	8	38	1.81
1OC0	0	--	--	0	--	--	0	--	--	0	--	--
1OPH	0	--	--	0	--	--	0	--	--	0	--	--
1OYV	8	12	1.62	8	12	1.62	7	3	1.79	7	24	1.59
BOYV	0	--	--	0	--	--	0	--	--	0	--	--
1PPE	35	1	0.79	35	1	0.79	37	1	1.22	40	1	0.65
1R0R	6	54	0.82	6	54	0.82	7	535	0.94	8	231	1.92
1TMQ	5	110	1.69	5	108	1.69	4	31	2.12	4	259	2.25
1UDI	3	26	2.00	3	25	2.00	2	515	2.44	3	32	2.31
1YVB	12	77	2.48	12	87	2.48	13	24	1.83	12	93	2.08
2ABZ	1	351	2.30	1	350	2.30	2	38	2.32	1	104	2.29
2B42	5	1	1.05	5	1	1.05	5	1	1.39	3	1	1.12
2J0T	0	2755	1.94	0	2787	1.94	1	1344	2.35	2	911	2.17
2MTA	0	--	--	0	--	--	1	986	2.16	0	2930	2.45
2O8V	0	--	--	0	--	--	0	--	--	0	--	--
2OUL	12	1	1.37	12	1	1.37	15	1	1.32	14	1	0.96
2PCC	0	--	--	0	--	--	1	1383	2.31	0	3048	2.31
2SIC	7	20	1.40	7	20	1.40	7	2	1.63	10	18	2.22
2SNI	1	1114	2.09	1	1098	2.09	3	386	1.94	1	1767	2.07
2UUY	16	15	2.22	16	15	2.22	18	173	2.43	16	48	2.48

3SGQ	6	79	2.09	6	79	2.09	8	38	1.59	6	75	2.50
7CEI	20	4	1.74	20	4	1.74	22	2	2.27	21	1	2.23
1A2K	0	--	--	0	--	--	0	--	--	0	--	--
1AK4	0	--	--	0	--	--	0	--	--	0	--	--
1AKJ	4	240	1.89	4	243	1.89	6	63	2.05	3	22	2.02
1AZS	10	52	2.19	11	57	2.19	11	108	1.38	9	130	2.25
1B6C	2	456	2.28	2	467	2.28	2	2	2.26	2	1363	2.33
1BUH	0	3211	1.79	0	3220	1.79	0	3022	1.61	0	--	--
1E96	1	365	2.01	1	364	2.01	1	441	1.90	0	--	--
1EFN	0	--	--	0	--	--	0	--	--	0	--	--
1F51	5	36	1.51	5	36	1.51	3	128	2.06	2	2	1.65
1FC2	0	--	--	0	--	--	0	--	--	0	--	--
1FCC	0	--	--	0	--	--	0	--	--	0	--	--
1FFW	0	--	--	0	--	--	0	--	--	0	--	--
1FQJ	0	--	--	0	--	--	0	--	--	0	2920	1.74
1GCQ	0	--	--	0	--	--	0	--	--	0	3193	2.04
1GHQ	0	--	--	0	--	--	0	--	--	0	--	--
1GLA	1	1160	2.26	1	1209	2.26	0	--	--	1	1033	2.10
1GPW	2	5	1.40	3	6	1.40	4	3	2.12	4	1	2.07
1H9D	0	--	--	0	--	--	0	--	--	0	--	--
1HCF	1	1964	1.89	1	1929	1.89	4	249	2.38	1	1736	2.33
1HE1	1	1844	2.14	1	1906	2.14	1	1197	2.16	1	1469	1.97
1I4D	0	--	--	0	--	--	0	--	--	0	--	--
1J2J	0	--	--	0	--	--	1	1374	2.42	0	--	--
1JWH	5	137	1.92	5	136	1.92	5	660	2.18	3	410	2.03
1K74	4	1	2.36	4	1	2.36	5	2	2.37	2	4	1.47
1KAC	1	347	2.50	1	347	2.50	1	946	2.31	0	--	--
1KLU	0	--	--	0	--	--	0	--	--	0	--	--
1KTZ	0	2456	2.24	0	2500	2.24	0	2610	1.33	1	1449	1.46
1KXP	4	8	2.35	4	9	2.35	4	3	2.09	4	12	2.14
1ML0	9	1	2.26	9	1	2.26	10	18	1.36	7	10	2.44
1OFU	2	1447	2.45	2	1420	2.45	2	875	2.43	2	34	2.42
1PVH	0	--	--	0	--	--	0	--	--	0	--	--
1QA9	0	2427	2.14	0	2410	2.14	0	--	--	0	--	--
1RLB	3	40	2.14	3	36	2.14	7	588	2.14	3	655	2.29
1RV6	0	--	--	0	--	--	0	--	--	0	--	--
1S1Q	1	973	2.12	1	966	2.12	1	735	1.19	1	152	1.78
1SBB	0	2633	1.11	0	2688	1.11	0	--	--	0	--	--
1T6B	2	167	1.91	2	159	1.91	2	236	1.58	3	330	2.23
1US7	0	--	--	0	--	--	0	--	--	1	642	1.58
1WDW	7	1	1.46	7	1	1.46	6	1	1.38	10	4	2.36
1XD3	2	604	2.16	2	545	2.16	1	1024	1.99	0	--	--
1XU1	2	998	1.86	2	1006	1.86	0	2691	1.58	1	1473	2.41
1Z0K	3	368	2.13	3	379	2.13	4	168	2.12	3	106	2.02
1Z5Y	0	--	--	0	2490	1.98	0	--	--	0	--	--
1ZHH	1	343	2.26	1	344	2.26	1	1828	1.95	1	1119	2.42
1ZHI	3	1141	1.84	3	1105	1.84	3	131	2.44	2	1613	1.91
2A5T	0	--	--	0	--	--	0	--	--	0	--	--
2A9K	0	--	--	0	--	--	2	255	2.35	0	--	--
2AJF	0	--	--	0	--	--	0	--	--	0	--	--
2B4J	0	--	--	0	--	--	0	--	--	0	2251	2.47
2BTF	0	2901	2.28	0	2889	2.28	0	--	--	0	2637	2.48
2FJU	0	--	--	0	--	--	0	--	--	0	--	--
2G77	1	1856	2.20	1	1775	2.20	2	140	2.24	1	1804	2.32
2HLE	4	7	2.16	4	7	2.16	1	11	2.13	2	31	2.39
2HQS	2	192	1.86	2	190	1.86	2	521	1.67	2	856	1.90
2OOB	0	--	--	0	--	--	0	--	--	0	--	--
2OOR	0	--	--	0	--	--	0	--	--	0	--	--
2VDB	1	476	1.32	1	479	1.32	3	129	0.93	3	460	0.75
3BP8	0	--	--	0	--	--	0	3135	1.91	0	3414	1.70
3D5S	9	1	0.95	9	1	0.95	6	8	1.70	9	54	0.82
1BGX	0	--	--	0	--	--	0	--	--	0	--	--
1ACB	0	--	--	0	--	--	0	--	--	0	--	--
1IJK	1	511	1.55	1	498	1.55	2	708	1.46	2	732	1.56

1JIW	0	--	--	0	--	--	0	--	--	0	--	--
1KKL	0	--	--	0	--	--	0	--	--	0	--	--
1M10	0	--	--	0	--	--	0	--	--	0	--	--
1NW9	0	--	--	0	--	--	0	--	--	0	--	--
4CPA	2	5	2.37	2	5	2.37	3	8	2.48	2	2	2.44
1GP2	0	--	--	0	--	--	0	--	--	0	--	--
1GRN	4	135	2.02	4	131	2.02	4	231	2.49	4	44	2.14
1HE8	0	--	--	0	--	--	0	--	--	0	--	--
1I2M	0	--	--	0	--	--	0	--	--	0	--	--
1IB1	0	--	--	0	--	--	0	--	--	0	--	--
1K5D	0	--	--	0	--	--	0	--	--	0	--	--
1LFD	1	1699	2.15	1	1754	2.15	1	830	2.38	1	707	2.22
1MQ8	2	186	2.30	2	194	2.30	1	505	2.50	1	735	2.41
1N2C	0	--	--	0	--	--	0	--	--	0	--	--
1R6Q	1	784	2.46	1	736	2.46	0	--	--	1	1977	2.44
1SYX	2	114	2.36	2	116	2.36	2	26	2.46	0	--	--
1WQ1	2	881	2.37	2	883	2.37	1	130	2.46	0	--	--
1XQS	1	897	2.47	1	781	2.47	1	246	2.20	1	778	2.06
2AYO	6	17	2.40	6	17	2.40	3	31	2.34	3	6	2.35
2CFH	3	7	2.08	3	7	2.08	4	8	1.82	5	18	1.75
2H7V	0	--	--	0	--	--	0	--	--	0	--	--
2HRK	0	--	--	0	--	--	0	--	--	0	--	--
2J7P	0	--	--	0	--	--	0	--	--	0	--	--
2NZ8	0	--	--	0	--	--	0	--	--	0	--	--
2OZA	0	--	--	0	--	--	0	--	--	0	--	--
2Z0E	0	--	--	0	--	--	0	--	--	0	--	--
3CPH	0	--	--	0	--	--	0	--	--	0	--	--
1E4K	0	--	--	0	--	--	0	--	--	0	--	--
2HMI	0	--	--	0	--	--	0	--	--	0	--	--
1F6M	0	--	--	0	--	--	0	--	--	0	--	--
1FQ1	0	--	--	0	--	--	0	--	--	0	--	--
1PXV	0	--	--	0	--	--	0	--	--	0	--	--
1ZLI	0	--	--	0	--	--	0	--	--	0	--	--
2O3B	0	--	--	0	--	--	0	--	--	0	--	--
1ATN	0	--	--	0	--	--	0	--	--	0	--	--
1BKD	0	--	--	0	--	--	0	--	--	0	--	--
1DE4	0	--	--	0	--	--	0	--	--	0	--	--
1EER	0	--	--	0	--	--	0	--	--	0	--	--
1FAK	0	--	--	0	--	--	0	--	--	0	--	--
1H1V	0	--	--	0	--	--	0	--	--	0	--	--
1IBR	0	--	--	0	--	--	0	--	--	0	--	--
1IRA	0	--	--	0	--	--	0	--	--	0	--	--
1JK9	0	--	--	0	--	--	0	--	--	0	--	--
1JMO	0	--	--	0	--	--	0	--	--	0	--	--
1JZD	0	--	--	0	--	--	0	--	--	0	--	--
1R8S	0	--	--	0	--	--	0	--	--	0	--	--
1Y64	0	--	--	0	--	--	0	--	--	0	--	--
1ZM4	0	--	--	0	--	--	0	--	--	0	--	--
2C0L	0	--	--	0	--	--	0	--	--	0	--	--
2I9B	0	--	--	0	--	--	0	--	--	0	--	--
2IDO	0	--	--	0	--	--	0	--	--	0	--	--
2OT3	0	--	--	0	--	--	0	--	--	0	--	--
