# Supplementary material for: Accelerating Protein Docking in ZDOCK Using an Advanced 3D Convolution Library
Source: PLoS One. 2011 Sep 19;6(9):e24657. doi: 10.1371/journal.pone.0024657 (PMC3176283; doi:10.1371/journal.pone.0024657)
Supplement: Table S2 — Predictive performance of ZDOCK 3.0, 3.0.1, 3.0.2f and 3.0.2 for the test cases in Benchmark 4.0. (PDF) [file pone.0024657.s002.pdf]

Table S2. Predictive performance of ZDOCK 3.0, 3.0.1, 3.0.2f and 3.0.2 for the test cases in Benchmark 4.0. Hits2K denotes the number of hits in the top 2000 predictions, Rank is the rank of first hit, and RMSD is the RMSD of first hit.

	ZDOCK 3.0			ZDOCK 3.0.1			ZDOCK 3.0.2f			ZDOCK 3.0.2		
**Test Case**	**Hits 2K**	**Rank**	**RMSD**	**Hits 2K**	**Rank**	**RMSD**	**Hits 2K**	**Rank**	**RMSD**	**Hits 2K**	**Rank**	**RMSD**
1AHW	7	182	2.37	7	177	2.37	7	137	2.35	3	386	1.71
1BVK	3	1018	2.38	3	1015	2.38	4	386	1.95	5	654	2.16
1DQJ	0	--	--	0	--	--	1	1622	1.83	2	1412	2.31
1E6J	16	10	2.31	16	9	2.31	13	11	2.22	13	3	2.42
1JPS	3	307	1.90	3	293	1.90	6	231	2.02	5	531	2.35
1MLC	9	486	1.15	9	488	1.15	11	312	1.78	10	71	1.54
1VFB	4	677	1.79	4	669	1.79	3	424	1.59	4	207	1.90
1WEJ	13	6	1.02	13	6	1.02	10	26	1.08	12	20	1.16
2FD6	5	25	2.09	6	24	2.09	7	10	1.37	7	39	2.13
2I25	18	1	2.06	18	1	2.06	18	8	2.44	18	2	1.75
2VIS	12	30	2.32	6	215	1.86	10	226	2.12	10	757	2.05
1BJ1	36	1	1.95	19	4	1.41	38	1	1.54	45	1	1.19
1FSK	17	4	0.91	17	4	0.91	18	2	1.99	14	6	2.28
1I9R	0	--	--	0	--	--	2	18	2.40	1	31	2.28
1IQD	13	5	1.12	13	5	1.12	14	2	2.50	11	2	1.75
1K4C	0	2337	1.08	0	2324	1.08	1	1828	1.06	2	926	1.26
1KXQ	3	35	1.30	3	35	1.30	3	107	2.01	4	6	1.16
1NCA	5	64	0.95	5	70	0.95	5	47	0.83	4	56	1.04
1NSN	0	--	--	0	--	--	1	652	0.91	1	1816	2.50
1QFW	3	443	1.36	4	450	1.36	3	596	1.42	3	801	1.28
2QFW	9	13	2.37	9	13	2.37	10	9	1.97	10	6	2.02
2JEL	18	9	1.53	18	9	1.53	23	19	1.48	21	13	1.53
1AVX	10	17	2.13	10	17	2.13	11	21	1.99	17	12	2.15
1AY7	3	1041	2.36	3	1042	2.36	5	1029	1.39	4	1117	2.29
1BVN	9	1	2.16	9	1	2.16	10	1	1.95	8	3	1.64
1CGI	2	261	2.47	2	257	2.47	2	120	2.45	2	11	2.43
1CLV	26	1	1.48	25	1	1.48	25	1	1.39	22	2	1.57
1D6R	0	--	--	0	--	--	0	--	--	0	--	--
1DFJ	4	1	1.93	4	1	1.93	5	1	2.03	6	1	1.90
1E6E	8	6	1.23	8	6	1.23	9	16	1.22	7	41	2.43
1EAW	8	83	1.31	9	83	1.31	9	66	1.73	7	51	1.49
1EWY	6	56	1.58	7	53	1.58	8	17	2.30	5	138	2.08
1EZU	5	302	2.03	3	320	2.03	6	335	2.48	4	396	1.61
1F34	2	68	2.37	2	59	2.37	1	503	1.98	2	312	1.78
1FLE	5	321	2.32	5	318	2.32	5	472	2.32	2	535	1.91
1GL1	6	16	1.44	6	16	1.44	6	84	1.57	3	54	1.46
1GXD	1	490	2.44	1	491	2.44	0	--	--	0	--	--
1HIA	0	--	--	0	--	--	0	--	--	0	--	--
1JTG	13	1	1.51	13	1	1.51	14	1	1.51	11	1	1.33
1MAH	14	1	1.01	14	1	1.01	14	1	1.24	15	1	1.02
1N8O	12	1	1.29	11	1	1.29	13	2	1.32	11	13	1.27
1OC0	0	--	--	0	--	--	0	--	--	1	190	2.21
1OPH	0	--	--	0	--	--	0	--	--	1	1358	2.41
1OYV	8	1	2.17	8	1	2.17	9	1	1.43	5	8	1.68
BOYV	0	--	--	0	--	--	0	--	--	0	--	--
1PPE	36	1	1.20	36	1	1.20	42	1	1.00	45	1	1.49
1R0R	8	66	0.97	8	65	0.97	9	98	1.05	8	173	2.48
1TMQ	11	15	1.74	11	15	1.74	11	13	2.15	9	9	1.90
1UDI	4	39	1.78	4	36	1.78	5	37	1.91	4	61	1.46
1YVB	20	4	1.99	19	4	1.99	18	2	2.08	20	1	1.74
2ABZ	3	86	2.15	3	84	2.15	1	280	2.11	1	421	2.20
2B42	6	3	1.63	6	3	1.63	6	2	1.55	7	4	2.17
2J0T	0	--	--	0	--	--	0	--	--	0	2895	2.49
2MTA	9	76	2.22	9	75	2.22	11	43	2.26	9	17	2.48
2O8V	2	412	2.48	2	414	2.48	1	1771	2.33	0	--	--
2OUL	13	1	1.32	13	1	1.32	16	1	1.17	15	1	1.51
2PCC	1	451	1.76	1	442	1.76	0	--	--	0	--	--
2SIC	13	3	0.75	13	3	0.75	14	1	1.28	18	2	1.34
2SNI	3	858	2.00	3	867	2.00	3	825	1.33	7	48	1.91
2UUY	8	312	2.29	8	311	2.29	10	492	1.84	11	374	1.83

3SGQ	5	294	1.94	5	296	1.94	3	321	2.23	5	292	1.18
7CEI	21	1	2.31	22	1	2.31	20	1	2.46	22	1	1.79
1A2K	0	2507	1.96	0	2545	1.96	0	--	--	1	1015	2.46
1AK4	0	--	--	0	--	--	0	2286	2.12	0	3298	2.46
1AKJ	3	145	1.86	2	282	1.89	3	156	1.91	1	1268	1.99
1AZS	26	3	1.32	15	4	2.09	27	3	1.42	20	4	1.61
1B6C	3	60	2.13	3	59	2.13	2	14	2.30	3	3	2.30
1BUH	9	9	2.37	9	8	2.37	7	100	1.84	7	5	2.08
1E96	1	588	2.13	1	557	2.13	1	267	2.09	1	1205	2.39
1EFN	0	2950	2.41	0	2945	2.41	0	2915	1.96	1	1701	2.42
1F51	8	1	1.84	0	2075	1.52	7	65	1.87	9	1	1.95
1FC2	0	--	--	0	--	--	0	--	--	0	--	--
1FCC	0	--	--	0	--	--	0	--	--	0	3518	2.50
1FFW	0	2502	2.15	0	2542	2.15	0	--	--	0	--	--
1FQJ	0	--	--	0	--	--	0	--	--	0	--	--
1GCQ	1	1169	1.05	1	1148	1.05	2	773	1.00	0	3197	2.45
1GHQ	0	--	--	0	--	--	0	--	--	0	--	--
1GLA	0	--	--	0	--	--	0	2021	2.36	4	698	2.33
1GPW	2	537	2.44	2	526	2.44	2	360	2.45	3	48	1.41
1H9D	4	163	1.94	4	157	1.94	3	303	2.14	2	252	2.49
1HCF	3	501	1.82	3	494	1.82	6	146	1.87	5	10	2.42
1HE1	1	440	1.86	1	429	1.86	1	478	1.93	2	20	2.30
1I4D	4	693	1.88	1	694	1.88	3	452	2.44	4	88	1.96
1J2J	3	141	1.90	3	142	1.90	6	50	2.49	2	42	2.18
1JWH	8	1	2.19	4	1	2.19	10	1	2.32	11	13	2.43
1K74	5	1	2.43	5	1	2.43	4	1	1.98	5	17	2.30
1KAC	2	624	2.46	2	619	2.46	1	1130	1.50	3	753	1.97
1KLU	1	1876	1.08	1	1917	1.08	0	3080	1.11	1	1795	1.41
1KTZ	0	2668	0.64	0	2691	0.64	0	2925	2.02	2	1310	1.09
1KXP	4	1	2.28	4	1	2.28	4	1	2.15	3	1	2.33
1ML0	8	13	1.65	6	13	1.65	9	10	2.06	13	1	1.85
1OFU	9	188	2.44	9	190	2.44	5	76	2.37	12	86	1.89
1PVH	6	1025	2.34	6	1023	2.34	2	1123	2.37	4	934	1.44
1QA9	0	--	--	0	--	--	0	--	--	0	--	--
1RLB	27	1	2.38	7	6	1.90	33	1	2.27	36	5	2.15
1RV6	15	3	1.53	9	3	1.53	16	1	1.68	11	3	2.16
1S1Q	2	1084	2.39	2	1075	2.39	2	968	1.86	3	936	1.57
1SBB	1	1542	1.94	1	1572	1.94	1	1762	1.94	1	1560	1.57
1T6B	2	309	1.91	2	333	1.91	4	42	1.68	3	166	2.45
1US7	2	255	2.38	2	251	2.38	1	344	1.46	2	181	1.25
1WDW	9	2	2.46	5	8	2.35	9	2	1.55	7	1	1.54
1XD3	12	15	2.42	12	15	2.42	11	24	2.44	10	7	2.32
1XU1	15	100	2.30	0	2260	2.32	12	123	2.36	12	104	2.44
1Z0K	4	20	1.63	4	20	1.63	5	1	1.75	4	2	1.94
1Z5Y	7	86	1.98	7	86	1.98	7	13	2.32	5	8	1.78
1ZHH	0	--	--	0	--	--	0	--	--	0	--	--
1ZHI	10	67	1.90	10	66	1.90	9	41	1.69	14	86	2.01
2A5T	0	--	--	0	--	--	0	--	--	0	--	--
2A9K	0	--	--	0	--	--	1	834	2.37	1	1602	1.91
2AJF	0	--	--	0	--	--	0	--	--	2	1015	2.34
2B4J	0	--	--	0	--	--	0	--	--	0	--	--
2BTF	6	65	1.06	6	67	1.06	6	241	2.09	5	459	2.21
2FJU	3	77	1.46	3	77	1.46	4	197	1.26	2	455	1.33
2G77	4	30	2.39	4	29	2.39	2	37	2.37	1	36	2.44
2HLE	3	91	2.32	3	84	2.32	4	12	2.26	3	281	2.47
2HQS	1	1701	2.19	1	1675	2.19	0	3244	2.37	0	--	--
2OOB	0	--	--	0	--	--	1	934	2.35	0	--	--
2OOR	0	--	--	0	--	--	1	161	2.45	0	--	--
2VDB	5	23	1.32	5	23	1.32	7	23	1.35	6	182	0.95
3BP8	3	276	1.51	1	1512	2.37	4	570	1.60	3	609	2.38
3D5S	7	56	1.95	7	58	1.95	5	85	1.66	6	73	1.73
1BGX	0	--	--	0	--	--	0	--	--	0	--	--
1ACB	0	--	--	0	--	--	0	--	--	0	--	--
1IJK	3	458	1.77	3	467	1.77	4	105	1.55	4	62	1.22

1JIW	0	--	--	0	--	--	0	--	--	0	--	--
1KKL	0	--	--	0	--	--	0	--	--	0	--	--
1M10	0	--	--	0	--	--	0	--	--	0	--	--
1NW9	0	--	--	0	--	--	0	--	--	0	--	--
4CPA	7	1	2.39	7	1	2.39	8	3	2.50	6	1	2.39
1GP2	0	--	--	0	--	--	0	--	--	0	--	--
1GRN	3	715	1.95	3	629	1.95	1	1462	2.37	3	841	1.94
1HE8	0	--	--	0	--	--	0	--	--	0	--	--
1I2M	1	219	2.39	1	210	2.39	1	184	2.43	1	128	2.49
1IB1	0	--	--	0	--	--	0	--	--	0	--	--
1K5D	1	1287	2.13	1	1483	2.13	0	--	--	2	180	2.36
1LFD	4	376	2.30	4	441	2.30	2	329	2.35	3	489	2.13
1MQ8	0	--	--	0	--	--	0	--	--	0	2061	2.37
1N2C	0	--	--	0	--	--	0	--	--	0	--	--
1R6Q	1	989	2.45	1	911	2.45	0	--	--	0	--	--
1SYX	0	--	--	0	--	--	1	1588	2.38	0	--	--
1WQ1	1	2	2.30	1	2	2.30	1	20	2.37	2	108	1.95
1XQS	3	44	2.09	3	39	2.09	2	18	2.07	2	34	2.25
2AYO	4	22	2.23	4	22	2.23	6	7	2.32	3	82	1.89
2CFH	7	2	2.21	7	2	2.21	7	1	2.23	6	1	1.82
2H7V	4	80	2.38	4	79	2.38	3	21	2.35	5	4	2.36
2HRK	1	232	2.49	1	231	2.49	3	61	2.45	0	--	--
2J7P	0	--	--	0	--	--	0	--	--	0	--	--
2NZ8	1	601	2.31	1	671	2.31	0	--	--	1	76	2.45
2OZA	0	--	--	0	--	--	0	--	--	0	--	--
2Z0E	0	--	--	0	--	--	0	--	--	0	--	--
3CPH	0	--	--	0	--	--	0	--	--	0	--	--
1E4K	0	--	--	0	--	--	0	--	--	0	--	--
2HMI	0	--	--	0	--	--	0	--	--	0	--	--
1F6M	0	--	--	0	--	--	0	--	--	0	--	--
1FQ1	0	--	--	0	--	--	0	--	--	0	--	--
1PXV	0	--	--	0	--	--	0	--	--	0	--	--
1ZLI	0	--	--	0	--	--	0	--	--	0	--	--
2O3B	0	--	--	0	--	--	0	--	--	0	--	--
1ATN	0	--	--	0	--	--	0	--	--	0	--	--
1BKD	0	--	--	0	--	--	0	--	--	0	--	--
1DE4	0	--	--	0	--	--	0	--	--	0	--	--
1EER	0	--	--	0	--	--	0	--	--	0	--	--
1FAK	0	--	--	0	--	--	0	--	--	0	--	--
1H1V	0	--	--	0	--	--	0	--	--	0	--	--
1IBR	0	--	--	0	--	--	0	--	--	0	--	--
1IRA	0	--	--	0	--	--	0	--	--	0	--	--
1JK9	0	--	--	0	--	--	0	--	--	0	--	--
1JMO	0	--	--	0	--	--	0	--	--	0	--	--
1JZD	0	--	--	0	--	--	0	--	--	0	--	--
1R8S	0	--	--	0	--	--	0	--	--	0	--	--
1Y64	0	--	--	0	--	--	0	--	--	0	--	--
1ZM4	0	--	--	0	--	--	0	--	--	0	--	--
2C0L	0	--	--	0	--	--	0	--	--	0	--	--
2I9B	0	--	--	0	--	--	0	--	--	0	--	--
2IDO	0	--	--	0	--	--	0	--	--	0	--	--
2OT3	0	--	--	0	--	--	0	--	--	0	--	--
