# Supplementary material for: Accelerating Protein Docking in ZDOCK Using an Advanced 3D Convolution Library
Source: PLoS One. 2011 Sep 19;6(9):e24657. doi: 10.1371/journal.pone.0024657 (PMC3176283; doi:10.1371/journal.pone.0024657)
Supplement: Table S3 — Running times of ZDOCK versions 2.3, 2.3.1, 2.3.2f, and 2.3.2 for the test cases in Benchmark 4.0. (PDF) [file pone.0024657.s003.pdf]

Table S3. Running time (in minutes) and running time fold improvement for new ZDOCK versions 2.3.1, 2.3.2f, and 2.3.2 (versus ZDOCK 2.3) for all test cases in docking Benchmark 4.0. L/R Switch indicates switching of ligand and receptor for that test case in ZDOCK 2.3.2.

	ZDOCK 2.3	ZDOCK 2.3.1		ZDOCK 2.3.2f		ZDOCK 2.3.2		
Test Case	Time	Time	Fold Improve	Time	Fold Improve	Time	Fold Improve	L/R Switch
1A2K	42.97	11.87	3.6	9.97	4.3	10.8	4.0	Y
1ACB	23.1	8.97	2.6	4.85	4.8	4.9	4.7	N
1AHW	93.58	23.27	4.0	20.93	4.5	17.63	5.3	Y
1AK4	31.4	8.72	3.6	9.88	3.2	6.38	4.9	Y
1AKJ	54.32	13.55	4.0	11.83	4.6	9.95	5.5	N
1ATN	51.87	11.93	4.3	10.73	4.8	9.98	5.2	N
1AVX	23.92	9.22	2.6	7.77	3.1	6.85	3.5	Y
1AY7	15.92	4.63	3.4	4.13	3.9	3.83	4.2	N
1AZS	58.23	17.48	3.3	17.02	3.4	13.28	4.4	Y
1B6C	40.68	15.25	2.7	12.95	3.1	7.52	5.4	Y
1BGX	127.4	38.32	3.3	31.17	4.1	22.97	5.5	Y
1BJ1	78.03	16.33	4.8	15.17	5.1	13.7	5.7	N
1BKD	92.98	13.7	6.8	11.47	8.1	11.5	8.1	N
1BUH	40.92	9.48	4.3	9.65	4.2	7.15	5.7	N
1BVK	31.25	10.03	3.1	9.28	3.4	6.45	4.8	N
1BVN	41.27	8.1	5.1	7.58	5.4	6.03	6.8	N
1CGI	24.52	4.98	4.9	4.57	5.4	4.62	5.3	N
1CLV	30.32	5.53	5.5	5.45	5.6	4.18	7.3	N
1D6R	23.87	7.17	3.3	5.07	4.7	4.72	5.1	N
1DE4	181.1	55.6	3.3	50.32	3.6	30.77	5.9	Y
1DFJ	54.78	16.32	3.4	14.05	3.9	9.78	5.6	Y
1DQJ	51.05	11.5	4.4	9.62	5.3	7.78	6.6	N
1E4K	76.12	18.42	4.1	14.93	5.1	12.02	6.3	N
1E6E	51	9.05	5.6	7.88	6.5	8.77	5.8	N
1E6J	41.42	9.43	4.4	7.05	5.9	6.83	6.1	N
1E96	42.43	12.13	3.5	11.27	3.8	9.88	4.3	Y
1EAW	18.3	5.3	3.5	4.62	4.0	4.63	4.0	N
1EER	80.38	25.52	3.1	24.8	3.2	14.5	5.5	Y
1EFN	13.63	3.82	3.6	2.92	4.7	2.92	4.7	N
1EWY	26.42	7.78	3.4	7.17	3.7	5.23	5.1	N
1EZU	44	13	3.4	13.03	3.4	9.25	4.8	Y
1F34	38.87	10.2	3.8	10.63	3.7	9.83	4.0	N
1F51	41.63	8.75	4.8	9.22	4.5	6.8	6.1	N
1F6M	40.18	8.1	5.0	8.8	4.6	6.02	6.7	N
1FAK	137.9	25.77	5.4	21.17	6.5	19.05	7.2	N
1FC2	53.4	14.93	3.6	14.65	3.6	8.4	6.4	Y
1FCC	40.85	7.97	5.1	7.23	5.7	6.75	6.1	N
1FFW	15.72	4.82	3.3	4.75	3.3	3.93	4.0	Y
1FLE	15.45	4.83	3.2	4.13	3.7	3.78	4.1	N
1FQ1	43.02	12.95	3.3	10.98	3.9	7.97	5.4	Y
1FQJ	50.48	13.03	3.9	9.98	5.1	9.8	5.2	N
1FSK	51.12	13.05	3.9	11.2	4.6	11.15	4.6	N
1GCQ	26.07	6.13	4.3	5.68	4.6	4.75	5.5	N
1GHQ	42.08	10.58	4.0	10.1	4.2	8.85	4.8	Y
1GL1	23.33	6.52	3.6	4.3	5.4	4.1	5.7	N
1GLA	50.53	11.22	4.5	9.88	5.1	8.35	6.1	N
1GP2	79.87	23.52	3.4	17.25	4.6	14.78	5.4	Y
1GPW	33.57	9.92	3.4	10.13	3.3	9.67	3.5	Y
1GRN	41.77	9.98	4.2	11.02	3.8	8.38	5.0	N
1GXD	77.62	18.57	4.2	13.98	5.6	13.2	5.9	N
1H1V	126.08	30.38	4.2	27.83	4.5	19.68	6.4	Y
1H9D	34.25	11.22	3.1	7.28	4.7	5.98	5.7	Y
1HCF	52.98	16.43	3.2	10.8	4.9	9.07	5.8	Y
1HE1	41.6	9.05	4.6	8.45	4.9	8.07	5.2	Y
1HE8	77.67	15.63	5.0	14.35	5.4	11.32	6.9	N
1HIA	18.45	5.33	3.5	4.65	4.0	4.7	3.9	N
1I2M	41.52	13.67	3.0	13.45	3.1	11.32	3.7	Y
1I4D	193.4	32.05	6.0	20.57	9.4	14.4	13.4	N

1I9R	58.02	19.95	2.9	19.75	2.9	14.42	4.0	Y
1IB1	77.32	18.27	4.2	12.53	6.2	11.55	6.7	N
1IBR	58.97	20.33	2.9	19.97	3.0	10.48	5.6	Y
1IJK	76.95	13.78	5.6	11.57	6.7	9.27	8.3	N
1IQD	53.97	12.73	4.2	11.27	4.8	10.25	5.3	N
1IRA	41.3	10.48	3.9	9.65	4.3	8.9	4.6	N
1J2J	23.52	4.58	5.1	4.5	5.2	4.57	5.1	N
1JIW	53.1	10.1	5.3	7.93	6.7	7.27	7.3	N
1JK9	32.8	9.72	3.4	9.28	3.5	6.23	5.3	N
1JMO	52.83	12.68	4.2	11.12	4.8	11.45	4.6	N
1JPS	90.18	20.95	4.3	19.27	4.7	17.8	5.1	Y
1JTG	33.07	10.28	3.2	8.37	4.0	8.78	3.8	N
1JWH	91.8	18.03	5.1	17.78	5.2	16.68	5.5	N
1JZD	55.5	14.3	3.9	12.15	4.6	10.58	5.2	N
1K4C	67.37	19.5	3.5	18.65	3.6	15.37	4.4	N
1K5D	77.48	21.67	3.6	16.8	4.6	14.97	5.2	Y
1K74	44.2	14.72	3.0	12.82	3.4	9.25	4.8	Y
1KAC	26.53	9.4	2.8	6.75	3.9	5.72	4.6	N
1KKL	41.28	7.93	5.2	7.88	5.2	6.98	5.9	N
1KLU	78.53	16.95	4.6	15.25	5.1	12.57	6.2	N
1KTZ	41.65	7.7	5.4	7.57	5.5	6.02	6.9	N
1KXP	97.03	25.53	3.8	23.47	4.1	18.68	5.2	Y
1KXQ	50.55	9.62	5.3	8.82	5.7	8.77	5.8	N
1LFD	15.93	5.27	3.0	4.95	3.2	5	3.2	N
1M10	77.88	20.5	3.8	14.47	5.4	10.68	7.3	Y
1MAH	30.75	9.38	3.3	8.17	3.8	7.73	4.0	N
1ML0	63.43	12.72	5.0	11.03	5.8	11.02	5.8	N
1MLC	51.07	12.33	4.1	9.85	5.2	7.52	6.8	N
1MQ8	53.55	9.15	5.9	8.9	6.0	7.33	7.3	N
1N2C	173.25	41.48	4.2	41.32	4.2	36.57	4.7	N
1N8O	31.45	11.62	2.7	8.82	3.6	7.42	4.2	Y
1NCA	79.38	18.63	4.3	18.4	4.3	15.13	5.2	N
1NSN	50.58	11.25	4.5	10.1	5.0	8.48	6.0	N
1NW9	33.97	8.97	3.8	7.88	4.3	5.82	5.8	N
1OC0	25.67	6.72	3.8	4.85	5.3	3.87	6.6	N
1OFU	39.95	11.58	3.4	11.52	3.5	10.3	3.9	Y
1OPH	54.27	12.55	4.3	11.2	4.8	11.08	4.9	N
1OYV	41	11.1	3.7	8.95	4.6	7.65	5.4	Y
1PPE	13.5	3.75	3.6	3.35	4.0	2.67	5.1	N
1PVH	64.53	14.65	4.4	11.58	5.6	8.62	7.5	N
1PXV	26.45	11.03	2.4	8.87	3.0	7.53	3.5	N
1QA9	118.75	20.85	5.7	16	7.4	15.33	7.7	N
1QFW	77.67	19.8	3.9	16.13	4.8	10.47	7.4	Y
1R0R	15.47	4.47	3.5	3.97	3.9	3.63	4.3	N
1R6Q	50.35	11.48	4.4	8.13	6.2	6.92	7.3	Y
1R8S	77.43	21.1	3.7	12.58	6.2	7.35	10.5	Y
1RLB	51.7	11.57	4.5	11.53	4.5	11.08	4.7	N
1RV6	32.35	6.83	4.7	6.33	5.1	5.98	5.4	N
1S1Q	19.27	4.7	4.1	4.02	4.8	3.48	5.5	N
1SBB	76.9	15.63	4.9	14.07	5.5	11.37	6.8	N
1SYX	18.45	5.05	3.7	3.95	4.7	3.35	5.5	N
1T6B	89.88	15.35	5.9	11.98	7.5	13.02	6.9	N
1TMQ	50.15	9.52	5.3	8.72	5.8	8.68	5.8	N
1UDI	24.12	8.63	2.8	6.67	3.6	4.88	4.9	N
1US7	42.07	10.82	3.9	10.1	4.2	7.28	5.8	Y
1VFB	40.58	10.5	3.9	10.28	3.9	6.6	6.1	N
1WDW	56.22	16.05	3.5	15.15	3.7	12.27	4.6	N
1WEJ	40.68	7.95	5.1	7.43	5.5	7	5.8	N
1WQ1	77.08	14.55	5.3	11.38	6.8	12.17	6.3	N
1XD3	15.62	4.65	3.4	4.38	3.6	4.52	3.5	N
1XQS	77.83	15.02	5.2	12.37	6.3	11.52	6.8	N
1XU1	30.15	8.25	3.7	8.22	3.7	6.45	4.7	N
1Y64	165.7	27.12	6.1	22.47	7.4	20.92	7.9	N

1YVB	38.25	10.15	3.8	7.68	5.0	7.73	4.9	N
1Z0K	23.63	6	3.9	4.47	5.3	4.28	5.5	N
1Z5Y	26.47	9.28	2.9	8.52	3.1	5.4	4.9	Y
1ZHH	53.85	13.82	3.9	13.6	4.0	11.37	4.7	N
1ZHI	53.02	10.82	4.9	9.7	5.5	7.78	6.8	N
1ZLI	41.52	9.78	4.2	7.48	5.6	6.03	6.9	N
1ZM4	166.97	22.43	7.4	21.08	7.9	17.35	9.6	N
2A5T	52.18	16.05	3.3	12.65	4.1	10.72	4.9	Y
2A9K	35.08	11.38	3.1	9.63	3.6	7.93	4.4	Y
2ABZ	23.05	6.18	3.7	4.93	4.7	4.63	5.0	N
2AJF	77.67	19.33	4.0	16.9	4.6	15.05	5.2	N
2AYO	41.25	7.67	5.4	6.5	6.3	7.18	5.7	N
2B42	42.1	10.65	4.0	7.83	5.4	8.07	5.2	N
2B4J	26.87	7.72	3.5	6.3	4.3	5.33	5.0	N
2BTF	40.97	10.33	4.0	8.25	5.0	7.12	5.8	N
2C0L	40.6	11.03	3.7	8.93	4.5	8.97	4.5	N
2CFH	32.82	9.48	3.5	8.78	3.7	6.45	5.1	Y
2FD6	57.38	16.02	3.6	15.9	3.6	14.23	4.0	N
2FJU	76.73	15.38	5.0	14.7	5.2	13.27	5.8	N
2G77	41.87	11.2	3.7	8.88	4.7	9.28	4.5	N
2H7V	93.5	15.4	6.1	13.57	6.9	11.9	7.9	N
2HLE	41.38	8.73	4.7	7.52	5.5	6.88	6.0	N
2HMI	140.35	34.6	4.1	30.4	4.6	26.15	5.4	N
2HQS	40.85	9.75	4.2	7.65	5.3	6.77	6.0	N
2HRK	50.22	11.57	4.3	7.1	7.1	6.35	7.9	N
2I25	23.35	8.55	2.7	6.08	3.8	5.42	4.3	N
2I9B	50.53	11.38	4.4	10.22	4.9	9.22	5.5	N
2IDO	25.92	8.13	3.2	6.57	3.9	5.9	4.4	Y
2J0T	24.18	9.42	2.6	7.42	3.3	5.63	4.3	Y
2J7P	78.63	17.98	4.4	14.4	5.5	14.47	5.4	N
2JEL	41	8.63	4.8	7.57	5.4	6.5	6.3	N
2MTA	38.3	9.78	3.9	9.75	3.9	7.7	5.0	N
2NZ8	54.13	14.45	3.7	13.03	4.2	10.97	4.9	N
2O3B	31.4	10.12	3.1	9.15	3.4	7.57	4.1	N
2O8V	24.55	7.9	3.1	5.77	4.3	5.12	4.8	N
2OOB	9.22	3.1	3.0	2.77	3.3	2.67	3.5	N
2OOR	77.12	14.18	5.4	13.85	5.6	12.68	6.1	N
2OT3	50.88	10.33	4.9	9.02	5.6	6.8	7.5	N
2OUL	41.1	9.95	4.1	9.83	4.2	7.32	5.6	N
2OZA	57.13	18.15	3.1	14.42	4.0	12.85	4.4	N
2PCC	23.55	9.05	2.6	7.92	3.0	6.03	3.9	N
2QFW	76.92	20.8	3.7	17.9	4.3	11.3	6.8	Y
2SIC	32.25	9.23	3.5	8.05	4.0	6.42	5.0	N
2SNI	23.9	6.55	3.6	5.1	4.7	4.83	4.9	N
2UUY	24.05	6.4	3.8	6.32	3.8	4.78	5.0	N
2VDB	54.7	13.28	4.1	8.9	6.1	8.23	6.6	N
2VIS	288.52	86.98	3.3	52.57	5.5	30.42	9.5	Y
2Z0E	32.9	10.2	3.2	9.12	3.6	7.45	4.4	N
3BP8	49.98	11.08	4.5	11.17	4.5	9.18	5.4	N
3CPH	50.98	12.77	4.0	11.07	4.6	9.87	5.2	N
3D5S	24.1	6.4	3.8	4.9	4.9	4.73	5.1	N
3SGQ	13.43	4.47	3.0	3.63	3.7	3.17	4.2	N
4CPA	25.38	6.18	4.1	4.67	5.4	4.4	5.8	N
7CEI	19.53	6.15	3.2	4.72	4.1	4.83	4.0	N
BOYV	41.43	11.23	3.7	9.37	4.4	7.68	5.4	Y
Average	53.20	13.13	4.0	11.23	4.7	9.32	5.5	
