# Supplementary material for: Accelerating Protein Docking in ZDOCK Using an Advanced 3D Convolution Library
Source: PLoS One. 2011 Sep 19;6(9):e24657. doi: 10.1371/journal.pone.0024657 (PMC3176283; doi:10.1371/journal.pone.0024657)
Supplement: Table S4 — Running times of ZDOCK versions 3.0, 3.0.1, 3.0.2f, and 3.0.2 for the test cases in Benchmark 4.0. (PDF) [file pone.0024657.s004.pdf]

Table S4. Running time (in minutes) and running time fold improvement for new ZDOCK versions 3.0.1, 3.0.2f, and 3.0.2 (versus ZDOCK 3.0) for all test cases in docking Benchmark 4.0. L/R Switch indicates switching of ligand and receptor for that test case in ZDOCK 3.0.2.

	ZDOCK 3.0	ZDOCK 3.0.1		ZDOCK 3.0.2f		ZDOCK 3.0.2		
Test Case	Time	Time	Fold Improve	Time	Fold Improve	Time	Fold Improve	L/R Switch
1A2K	130.8	21.37	6.1	20.05	6.5	21.45	6.1	Y
1ACB	73.35	17.38	4.2	9.97	7.4	10	7.3	N
1AHW	302.63	50.28	6.0	45.5	6.7	35.22	8.6	Y
1AK4	96.75	21.25	4.6	21.15	4.6	12.48	7.8	Y
1AKJ	170.65	23.48	7.3	23.47	7.3	18.83	9.1	N
1ATN	165.6	20.62	8.0	20.68	8.0	19.33	8.6	N
1AVX	72.17	16.57	4.4	15.67	4.6	12.87	5.6	Y
1AY7	48.52	8.42	5.8	8.4	5.8	7.55	6.4	N
1AZS	178.35	35.17	5.1	34.5	5.2	25.58	7.0	Y
1B6C	122.18	27.28	4.5	25.95	4.7	15.23	8.0	Y
1BGX	391.65	66.13	5.9	60.32	6.5	46.72	8.4	Y
1BJ1	248.68	33.72	7.4	32.22	7.7	28.57	8.7	N
1BKD	296.85	24.5	12.1	22.75	13.0	23.73	12.5	N
1BUH	131.17	19.13	6.9	21.12	6.2	15.12	8.7	N
1BVK	103.12	19.97	5.2	19.5	5.3	12.83	8.0	N
1BVN	130.12	16	8.1	16.1	8.1	12.32	10.6	N
1CGI	76.7	9.7	7.9	9.53	8.0	9.57	8.0	N
1CLV	97.93	11.73	8.3	11.87	8.3	8.58	11.4	N
1D6R	72.8	13.97	5.2	10.88	6.7	9.97	7.3	N
1DE4	541.57	99.28	5.5	98.7	5.5	62.07	8.7	Y
1DFJ	166.35	27.83	6.0	26.32	6.3	21.15	7.9	Y
1DQJ	163.82	24.07	6.8	20.72	7.9	15.5	10.6	N
1E4K	211.43	37.28	5.7	31.72	6.7	24.98	8.5	N
1E6E	164.33	18.43	8.9	15.82	10.4	18.35	9.0	N
1E6J	128.77	16.72	7.7	14.75	8.7	14.43	8.9	N
1E96	130.3	22.88	5.7	23.9	5.5	20.82	6.3	Y
1EAW	57.82	10.23	5.7	9.52	6.1	9.53	6.1	N
1EER	249.03	53.42	4.7	53.75	4.6	29.82	8.4	Y
1EFN	42.25	7.03	6.0	5.8	7.3	5.8	7.3	N
1EWY	83.65	13.22	6.3	14.93	5.6	10.2	8.2	N
1EZU	133.22	25.75	5.2	25.73	5.2	17.53	7.6	Y
1F34	125.73	21.43	5.9	22.47	5.6	20	6.3	N
1F51	131.18	17.98	7.3	19.07	6.9	13.8	9.5	N
1F6M	131.28	16.23	8.1	17.97	7.3	11.78	11.1	N
1FAK	449.83	53.73	8.4	47.03	9.6	41.22	10.9	N
1FC2	163.65	28.25	5.8	28.03	5.8	18.33	8.9	Y
1FCC	128.48	16.82	7.6	15.25	8.4	14.38	8.9	N
1FFW	48.32	9.88	4.9	9.85	4.9	7.17	6.7	Y
1FLE	47.88	10.2	4.7	8.53	5.6	7.68	6.2	N
1FQ1	127.77	22.32	5.7	21.13	6.0	15.8	8.1	Y
1FQJ	163.5	27.43	6.0	21.32	7.7	19.78	8.3	N
1FSK	172.45	23.9	7.2	22.78	7.6	22.28	7.7	N
1GCQ	83.77	12.52	6.7	11.78	7.1	9.7	8.6	N
1GHQ	132.57	21.93	6.0	21.93	6.0	16.3	8.1	Y
1GL1	77.22	13.57	5.7	9.23	8.4	8.53	9.1	N
1GLA	163.43	23.45	7.0	20.5	8.0	16.45	9.9	N
1GP2	249.3	49.55	5.0	34.5	7.2	29.67	8.4	Y
1GPW	102.55	20.22	5.1	20.08	5.1	18.88	5.4	Y
1GRN	131.43	22.62	5.8	23.1	5.7	16.72	7.9	N
1GXD	245.43	39.5	6.2	29.47	8.3	26.88	9.1	N
1H1V	394.47	58.87	6.7	54.2	7.3	40.33	9.8	Y
1H9D	96.85	24.42	4.0	14.78	6.6	12.08	8.0	Y
1HCF	167.78	43.17	3.9	23.65	7.1	18.2	9.2	Y
1HE1	133.25	16.73	8.0	16.67	8.0	16.8	7.9	Y
1HE8	246.88	32.1	7.7	30.05	8.2	22.37	11.0	N
1HIA	58.45	11.48	5.1	10.05	5.8	10.05	5.8	N
1I2M	124.03	25.58	4.8	25.1	4.9	22.82	5.4	Y
1I4D	655.48	67.83	9.7	44.1	14.9	30.15	21.7	N

1I9R	177.07	40.15	4.4	39.83	4.4	26.4	6.7	Y
1IB1	249.17	38.68	6.4	25.88	9.6	24.27	10.3	N
1IBR	179.38	47.52	3.8	47.12	3.8	20.6	8.7	Y
1IJK	255.88	24.77	10.3	23.33	11.0	18.35	13.9	N
1IQD	168.5	27.08	6.2	23.3	7.2	21.65	7.8	N
1IRA	131.8	21.07	6.3	19.55	6.7	17.6	7.5	N
1J2J	71.75	9.77	7.3	9.6	7.5	9.88	7.3	N
1JIW	167.97	20.42	8.2	16.25	10.3	14.82	11.3	N
1JK9	98.33	18.43	5.3	19.32	5.1	12.35	8.0	N
1JMO	170.5	21.35	8.0	20.97	8.1	22.37	7.6	N
1JPS	300.15	45.13	6.7	42.87	7.0	35.23	8.5	Y
1JTG	96.92	19.9	4.9	17.13	5.7	18.02	5.4	N
1JWH	300.35	36.07	8.3	36.07	8.3	33.25	9.0	N
1JZD	177.93	30.28	5.9	25.73	6.9	22.2	8.0	N
1K4C	209.68	43.18	4.9	37.55	5.6	30.12	7.0	N
1K5D	211.75	35.38	6.0	34.25	6.2	29.13	7.3	Y
1K74	127.73	28.75	4.4	25.35	5.0	17.82	7.2	Y
1KAC	83.27	19.88	4.2	13.83	6.0	11.45	7.3	N
1KKL	127.43	16.78	7.6	16.73	7.6	14.48	8.8	N
1KLU	211.43	31.53	6.7	31.03	6.8	24.78	8.5	N
1KTZ	131.93	16	8.2	15.83	8.3	12.57	10.5	N
1KXP	299.08	52.32	5.7	47.73	6.3	37.67	7.9	Y
1KXQ	165	17.2	9.6	17.53	9.4	18.45	8.9	N
1LFD	47.87	9.98	4.8	9.92	4.8	9.95	4.8	N
1M10	214.15	39.95	5.4	30.72	7.0	22.25	9.6	Y
1MAH	96.25	19.58	4.9	17.2	5.6	16.32	5.9	N
1ML0	211.57	27.32	7.7	23.2	9.1	22.85	9.3	N
1MLC	162.38	22.67	7.2	20.57	7.9	14.9	10.9	N
1MQ8	171.08	18.38	9.3	17.87	9.6	14.48	11.8	N
1N2C	447.17	85.8	5.2	85.8	5.2	70.08	6.4	N
1N8O	96.95	24.55	3.9	17.82	5.4	13.95	6.9	Y
1NCA	247.35	36.7	6.7	36.52	6.8	28.43	8.7	N
1NSN	164.3	22.7	7.2	20.95	7.8	16.65	9.9	N
1NW9	102.45	18.35	5.6	16.4	6.2	11.72	8.7	N
1OC0	83.05	14.2	5.8	10.2	8.1	7.87	10.6	N
1OFU	123.67	22.48	5.5	22.42	5.5	19.73	6.3	Y
1OPH	170.48	22.08	7.7	21.77	7.8	22.73	7.5	N
1OYV	132.18	19.45	6.8	19.13	6.9	14.58	9.1	Y
1PPE	42.35	7.95	5.3	7.13	5.9	5.53	7.7	N
1PVH	210.5	26.82	7.8	23.57	8.9	17.53	12.0	N
1PXV	83.25	19.17	4.3	19.12	4.4	15.65	5.3	N
1QA9	366.82	39.78	9.2	34.42	10.7	33.55	10.9	N
1QFW	248.37	43.68	5.7	35.22	7.1	21.15	11.7	Y
1R0R	48.75	9.13	5.3	8.12	6.0	7.4	6.6	N
1R6Q	162.98	25.33	6.4	17.97	9.1	14.27	11.4	Y
1R8S	256.1	47.28	5.4	27.38	9.4	14.37	17.8	Y
1RLB	164	23.63	6.9	23.27	7.0	22.33	7.3	N
1RV6	102.83	13.78	7.5	12.93	8.0	12.08	8.5	N
1S1Q	60.78	8.77	6.9	8.17	7.4	6.83	8.9	N
1SBB	209.97	29.2	7.2	28.9	7.3	22.55	9.3	N
1SYX	58.42	9.6	6.1	7.92	7.4	6.53	8.9	N
1T6B	294.28	31.05	9.5	23.43	12.6	25.72	11.4	N
1TMQ	163.02	18.18	9.0	18	9.1	18.47	8.8	N
1UDI	76.18	13.77	5.5	13.75	5.5	9.93	7.7	N
1US7	132.78	23	5.8	22.18	6.0	13.78	9.6	Y
1VFB	131.48	22.9	5.7	22.07	6.0	13.42	9.8	N
1WDW	176.87	31.88	5.5	30.28	5.8	23.9	7.4	N
1WEJ	131.13	14.9	8.8	14.68	8.9	14.08	9.3	N
1WQ1	247.18	30.22	8.2	23.1	10.7	25.85	9.6	N
1XD3	47.78	8.63	5.5	8.6	5.6	9.3	5.1	N
1XQS	248.93	29.82	8.3	25.83	9.6	24.17	10.3	N
1XU1	96.62	17.87	5.4	18.03	5.4	13.75	7.0	N
1Y64	538.68	56.03	9.6	45.82	11.8	43.4	12.4	N

1YVB	122.52	18.7	6.6	16.3	7.5	16.15	7.6	N
1Z0K	75.87	10.43	7.3	9.52	8.0	8.92	8.5	N
1Z5Y	84.17	16.98	5.0	18.43	4.6	10.35	8.1	Y
1ZHH	169.28	28.25	6.0	28.2	6.0	22.98	7.4	N
1ZHI	171.62	21.17	8.1	21.25	8.1	16.18	10.6	N
1ZLI	132.5	21.22	6.2	16.2	8.2	12.28	10.8	N
1ZM4	528.27	51.9	10.2	45.38	11.6	36.57	14.4	N
2A5T	166.07	28.73	5.8	24.88	6.7	20.65	8.0	Y
2A9K	102.87	22.73	4.5	19.22	5.4	15.58	6.6	Y
2ABZ	72.27	11.92	6.1	10.13	7.1	9.38	7.7	N
2AJF	250.25	41.23	6.1	35.83	7.0	31.68	7.9	N
2AYO	132.77	16.17	8.2	13.22	10.0	15.32	8.7	N
2B42	126.72	20.98	6.0	15.28	8.3	15.48	8.2	N
2B4J	82.97	12.98	6.4	13.1	6.3	10.95	7.6	N
2BTF	128.9	18.38	7.0	16.93	7.6	14	9.2	N
2C0L	131.3	23.4	5.6	19.3	6.8	18.43	7.1	N
2CFH	98.43	19.3	5.1	18.7	5.3	12.68	7.8	Y
2FD6	179.37	32.77	5.5	32.32	5.5	28.57	6.3	N
2FJU	211.45	30.58	6.9	30.68	6.9	27.32	7.7	N
2G77	131.15	22.78	5.8	17.82	7.4	18.97	6.9	N
2H7V	302.15	31.9	9.5	28.27	10.7	25.9	11.7	N
2HLE	131.25	15.35	8.6	15.5	8.5	13.83	9.5	N
2HMI	436.12	70.43	6.2	63.13	6.9	52.8	8.3	N
2HQS	128.33	17.4	7.4	15.92	8.1	13.53	9.5	N
2HRK	164.03	21.82	7.5	15.17	10.8	13.22	12.4	N
2I25	72.12	15.35	4.7	12.37	5.8	10.58	6.8	N
2I9B	165.67	24.33	6.8	21.22	7.8	19.15	8.7	N
2IDO	82.68	16.38	5.0	14.3	5.8	11.1	7.4	Y
2J0T	76.57	19.42	3.9	15.02	5.1	10.65	7.2	Y
2J7P	248.77	37.7	6.6	29.62	8.4	30	8.3	N
2JEL	134.63	17.53	7.7	15.15	8.9	13.02	10.3	N
2MTA	121.42	20.62	5.9	20.77	5.8	15.95	7.6	N
2NZ8	171.95	35.43	4.9	27.87	6.2	22.8	7.5	N
2O3B	97.75	21.75	4.5	19.48	5.0	15.87	6.2	N
2O8V	72.08	13.82	5.2	11.65	6.2	10.08	7.2	N
2OOB	27.75	5.5	5.0	5.47	5.1	5.13	5.4	N
2OOR	247.9	30.63	8.1	29.63	8.4	26.8	9.3	N
2OT3	162.97	20.2	8.1	17.67	9.2	12.8	12.7	N
2OUL	131.03	21.37	6.1	21.1	6.2	15.17	8.6	N
2OZA	180.57	43.1	4.2	29.03	6.2	25.3	7.1	N
2PCC	72.6	18.77	3.9	16.27	4.5	11.93	6.1	N
2QFW	211.6	43.72	4.8	39.08	5.4	25.67	8.2	Y
2SIC	97.98	18.58	5.3	17.12	5.7	13.2	7.4	N
2SNI	75.78	12.68	6.0	10.48	7.2	10.05	7.5	N
2UUY	77.05	13.85	5.6	13.77	5.6	10.32	7.5	N
2VDB	173.82	28.68	6.1	18.83	9.2	17.48	9.9	N
2VIS	923.4	199.17	4.6	111.28	8.3	62.8	14.7	Y
2Z0E	98.17	21.38	4.6	19.03	5.2	15.17	6.5	N
3BP8	169.27	23.27	7.3	22.9	7.4	19.37	8.7	N
3CPH	164.98	26.02	6.3	22.5	7.3	19.1	8.6	N
3D5S	72.48	13.25	5.5	10.1	7.2	9.63	7.5	N
3SGQ	43.07	9.2	4.7	7.48	5.8	6.53	6.6	N
4CPA	84.55	12.72	6.6	9.98	8.5	9.28	9.1	N
7CEI	60.27	11.38	5.3	8.98	6.7	9.27	6.5	N
BOYV	130.48	24.68	5.3	20.55	6.3	14.5	9.0	Y
Average	166.64	26.48	6.3	23.24	7.1	18.86	8.6	
